# The Overseas Transport of Wounded: Life and Procedure on a Hospital Ship
*The following article is available by kind permission of the Officer Commanding Ambulance Ship *St. David.*


**Published:** 1915-04-17

**Authors:** 


### THE OVERSEAS TRANSPORT OF WOUNDED.

### Life and Procedure on a Hospital Ship.*

By A MEDICAL OFFICER ON THE AMBULANCE SHIP "ST. DAVID."

The position of medical officer on board the ships now in use for overseas transport of wounded is one of the most interesting on active service; he is on the last lines of communication of the British Expeditionary Force, he sees the aftermath of trench warfare, and literally has his finger on the pulses of those whose wounds necessitate a journey home. The British public have little knowledge of the means taken to ensure the comfort and ease of the wounded *en voyage*, and the precautions for their safety. Great improvement has taken place in recent months in the overseas transport, and now it is at a very high state of efficiency—a tribute which the wounded would be the first to pay.

### Preparations.

The usual procedure now is for the cases for transport to be selected and sorted at the base hospitals—rarely directly from the ambulance trains—and, with the good offices of an official medical embarkation officer and his staff, to be conveyed to the hospital ship. The embarkation officer makes arrangements and is responsible for the quayside details of transference. He decides on the number of the convoy, the division into "cot" and "sitting" cases; telephones his requirements to the base hospitals, and occasionally he has to take "batches" from various hospitals; appoints a definite time for the transference—after consultation with the naval transport officer as to tides and the desirability of departure; orders the necessary quota of motor ambulances, and gives instructions to his staff of stretcher-bearers—in fact, the hundred-and-one small, important, and worrying details, attention to which makes for the smooth running of the transfer, are in his hands; and he has all his time fully occupied, not only with the embarkation of cases to hospital ships, but with the disembarkation of ambulance trains from the Front and the removal of their tale of cases to the base hospitals.

### The Nominal Roll.

When all arrangements are complete and the official routine met the order is given, and from the deck of the hospital ship all that is seen is a long streak of Red Cross motor ambulances threading their way quickly and busily through the streets of the port. Of the actual worry of transference at the base hospitals and its crowded details we on board know little. Once on the quay, the cases—usually four stretcher cases in each ambulance or a number of sitting cases—are transferred by bearers to the ships. The sitting cases walk on; the stretcher cases are simply removed in the same stretcher from the ambulance to the ship by two or four orderlies. Tidal occurrences often cause difficulties with the ship's gangway, but the ingenuity of the mercantile marine is usually equal to the emergency.

### In the Wards.

Once on board the ship, however, our procedure holds: each case is received at the gangway by a non-commissioned officer, with assistants, who keeps a tally of the total cases. The sitting cases are directed to their part of the ship, but before being allotted their seats in their room each case has his name and regiment and other particulars taken for the purposes of the *Nominal Roll*—a most important document by which cases can be traced *en transport*, and often required in duplicate or triplicate. The lying-down cases, or stretcher cases, are allotted to their respective wards. The medical officers are stationed in their wards, receive the cases there, decide on the appropriate bed, and see to the proper and careful removal from stretcher to bed, in which process the embarkation orderlies co-operate with the orderlies of the ship. In this way all the beds are filled up. The officers, sitting or lying down, are conducted to their special ward, but on occasions it has been found necessary to put a small overflow of men in their wards or *vice versa*.

### The Medical Inspection.

While this embarkation is still proceeding the nursing sister of each ward attends to the patient. His name, regiment, wound, and other particulars are taken for the Ward Book and for the Nominal Roll; the patient is made comfortable with pillows and bed-rests; inquiries are made as to the time of his last meal and such other items; his pulse and temperature are taken. Everything is held in readiness for the medical officer, who pays a formal visit at the end of the embarkation, urgent cases being, of course, attended to at any time. The medical officer on his visit gets a hurried verbal history of each case; only in very special cases are histories sent from the base hospitals. He decides treatment necessary—dressing or no dressing; drugs or no drugs; stimulants or analgesics; his instructions are usually in writing.

The actual amount of medical attention or surgical work depends on a variety of conditions—viz., the length of time the patient remains on board between embarkation and departure, the nature of the wound, the distress or discomfort of the patient, the probable length of the sea journey, the nature of the weather and railway journey on the English side, and other points. Every aspect is carefully considered, the paramount considerations being the comfort, ease, and well-being of the wounded. The sister and ward orderlies are always in attendance. If it is necessary for patients to be

* The following article is available by kind permission of the Officer Commanding Ambulance Ship *St. David*.

on board overnight, as frequently occurs, one sister is apportioned to this night duty alternately, with an orderly to each ward.

Administrative Work on the Voyage.

A considerable amount of administrative and clerical work has to be done during the voyage: the *Disembarkation State*, differentiating the types of cases, the Nominal Roll for special and general cases, the case sheets and particulars of special cases (when available), and a return of valuables of prisoners or mental cases have all to be made out, perhaps in a short voyage of only two hours; Red Cross postcards to tell relatives of their ultimate destination to be distributed; officers' kits to be labelled and numbered; the cases to be examined by the medical officers, to decide the Red Labels, viz. those unable to stand a long railway journey, and the White Labels, viz. those able to travel any distance; mental cases to be searched for weapons. This ought properly to be done on shore, but is a wise precaution in any case. On disembarkation the process is reversed: the ship's orderlies remove the cases, under the supervision of the medical officer, to the stretchers; this is done in a regular order. The embarkation orderlies remove the stretcher patients, well covered and warmly clad, to the station platform and luxuriously fitted English ambulance trains, and so on to English hospitals.

A great number of the administrative difficulties of a hospital ship have still to be mentioned: only those that relate to the patients have been touched on above; but such questions as the cleansing and washing of the ship after each convoy, the commissariat and laundry arrangements, have all to be carefully considered.

The Route and the Enemy.

On the present overseas route, or the changes of route, no comment will be allowed, though from conversations with returned wounded many of the public are in possession of our ports of call and our routes. And it is a fairly safe surmise that the enemy is possessed of full knowledge of our movements. According to the Hague Conventions, the enemy must be informed of the name and other details of every ship in use or commission for overseas transport of wounded. This requirement, it is safe to say, has been carried out, so that the enemy know of every ship thus transformed.

There are, at present, probably about eighteen hospital ships in use, but they are of different types, and call at different ports. The writer, probably biassed from six months' sojourn in one of them, prefers the three "Saints" ambulance ships. Such ships, formerly passenger ships on the Fishguard-Rosslare route, are of a convenient size, suitable draught, and sufficient speed (twenty-two knots per hour). The average number in a convoy is about 250 to 270, but considerations of weather, length of voyage, time of year, and nature of wounds may modify the circumstances or lessen the number.

Mal-de-Mer and Other Symptoms.

The treatment varies. It may be taken for granted that every case has his wounds dressed daily if on board, or more frequently if necessary. The factor of *mal-de-mer* has to be alleviated by suitable drug treatment and posture; the discomforts of splintage, and jolting of fractured bony ends to be overcome. The care of those sick, not wounded; the transference of a case from one type to another, say, from a sitting case to a lying-down case, on board ship, has to be attended to. This may only be done after careful medical examination and inspection of wounds. The constant guard against infection, and the look-out for incipient symptoms of tetanus, are striking features of the work. As aseptic surgery is impossible, antisepsis holds the field. There are electric sterilisers in each ward, to ensure the asepticity of instruments. The ships are not luxuriously fitted out, or extravagantly equipped, but they serve their purpose of efficiency and utility with comfort to the wounded. Morphine, here as elsewhere, is a standby.

The Life of the Staff.

The life, though necessarily cramped and somewhat unhealthy, is a most interesting one for the medical *personnel*. The staff comprises a commanding officer (lieutenant-colonel), with three subordinate medical officers, four nursing sisters, and four non-commissioned officers and twenty-two orderlies. The amusement of the patients has not been neglected, and, through the kindness and generosity of private individuals and one of the ship's officers, gramophones with suitable records have enabled the monotony of the voyage to be forgotten.

The life day by day is of unfailing interest because of incidents *en voyage*, the comments and stories of the wounded, the contrast afforded between different ports and different national peculiarities, and a first-hand knowledge of how the wounded fare, from the time of the wound infliction till their arrival in England, with the opinions of the wounded themselves on the various stages. Tommy Atkins, even when wounded, is a very cheery soul, and makes a most pleasant patient.

Many and many a stirring story could easily be woven round the vivid descriptions of fighting incidents by the wounded; many a tale could be recorded of incidents *en voyage*, or "happenings" in a French port, or rumoured accounts of impossible deeds. Many a psychological study full of human pathos or humour, or absorbing interest, could be undertaken and narrated. Every item would occupy several pages: every administrative detail would need a chapter. Hospital ships—ambulance or otherwise—are comparatively new accessories to modern warfare; they are at present in course of evolution; and this European war will establish in this respect many a precedent, and probably necessitate a special set of official regulations in the military annals.

